# Supplementary material for: Personalized Nutritional Strategies to Reduce Knee Osteoarthritis Severity and Ameliorate Sarcopenic Obesity Indices: A Practical Guide in an Orthopedic Setting
Source: Nutrients. 2023 Jul 9;15(14):3085. doi: 10.3390/nu15143085 (PMC10385346; doi:10.3390/nu15143085)
Supplement: Supplementary file 1 [file nutrients-15-03085-s001.zip › nutrients-2484014-supplementary.pdf]

Academy of Nutrition and Dietetics

Narrative Review Checklist

Section/topic	#	Checklist item	Reported on page or line #
**TITLE**			
Title	1	Identify the report as a Narrative Review of …	
**ABSTRACT**			
Unstructured summary	2	Provide an unstructured summary including, as applicable: background, objective, brief summary of narrative review and implications for future research, and clinical practice or policy development.	
**INTRODUCTION**			
Rationale/background	3	Describe the rationale for the review in the context of what is already known.	
Objectives	4	Specify the key question(s) identified for the review topic.	
**METHODS**			
Research selection	5	Specify the process for identifying the literature search (eg, years considered, language, publication status, study design, and databases of coverage).	
**DISCUSSION/SUMMARY**			
Narrative	6	Discuss: 1) research reviewed including fundamental or key findings, 2) limitations and/or quality of research reviewed, and 3) need for future research.	
Summary	7	Provide an overall interpretation of the narrative review in the context of clinical practice and/or the Nutrition Care Process for registered dietitian nutritionists, clinical practice for other health professionals, policy development and implementation, or future research.	
